# Identification of Postoperative Prognostic MicroRNA Predictors in Hepatocellular Carcinoma

**DOI:** 10.1371/journal.pone.0037188

**Published:** 2012-05-22

**Authors:** Ya-Hui Huang, Kwang-Huei Lin, Hua-Chien Chen, Ming-Ling Chang, Chao-Wei Hsu, Ming-Wei Lai, Tse-Ching Chen, Wei-Chen Lee, Yi-Hsin Tseng, Chau-Ting Yeh

**Affiliations:** 1 Department of Hepato-Gastroenterology, Liver Research Center, Chang Gung Memorial Hospital, Taoyuan, Taiwan; 2 Department of Biochemistry, School of Medicine, Chang Gung University, Taoyuan, Taiwan; 3 Department of Life Science, Chang Gung University, Taoyuan, Taiwan; 4 Department of Pediatric Gastroenterology, Chang Gung Children Hospital, Taoyuan, Taiwan; 5 Department of Pathology, Department of Surgery, Chang Gung Memorial Hospital, Taipei, Taiwan; 6 Division of General Surgery, Department of Surgery, Chang Gung Memorial Hospital, Taipei, Taiwan; 7 Molecular Medicine Research Center, Chang Gung University, Taoyuan, Taiwan; University of Hong Kong, Hong Kong

## Abstract

Comparison of microRNA (miRNA) expression profiles in the noncancerous liver tissues adjacent to hepatocelluar carcinomas (HCCs) was a strategy to identify postoperative prognostic predictors in this study. Expression profiles of 270 miRNAs were determined in the paraneoplastic liver tissues of 12 HCC patients with known postoperative prognosis. A panel of candidate miRNA predictors was identified. The prognostic predictive value of these candidate miRNAs was then verified in 216 postoperative HCC patients. Univariate analysis identified 8 and 3 miRNA predictors for recurrence-free (RFS) and overall (OS) survivals, respectively. Multivariate analysis revealed high expression levels of miR-155 (HR, 2.002 [1.324–3.027]; *P* = .001), miR-15a (HR, 0.478 [0.248–0.920]; *P* = .027), miR-432 (HR, 1.816 [1.203–2.740]; *P* = .015), miR-486-3p (HR, 0.543 [0.330–0.893]; *P* = .016), miR-15b (HR, 1.074 [1.002–1.152]; *P* = .043) and miR-30b (HR, 1.102 [1.025–1.185]; *P* = .009) were significantly associated with RFS. When clinicopathological predictors were included, multivariate analysis revealed that tumor number and miR-432, miR-486-3p, and miR-30b expression levels remained significant as independent predictors for RFS. Additionally, expression knockdown of miR-155 in J7 and Mahlavu hepatoma cells resulted in decreased cell growth and enhanced cell death in xenograft tumors, suggesting an oncogenic effect of miR-155. In conclusion, significant prognostic miRNA predictors were identified through examination of miRNA expression levels in paraneoplastic liver tissues. Functional analysis of a miRNA predictor, miR-155, suggested that the prognostic miRNA predictors identified under this strategy could serve as potential molecular targets for anticancer therapy.

## Introduction

Hepatocellular carcinoma (HCC) is the fifth most common solid malignant tumors worldwide and the third leading cause of cancer related death. Hepatitis viruses infection (hepatitis B or C) and long-term ingestion of some chemical agents (alcohol or aflatoxin B1) are the known etiological factors for the occurrence of HCC [1]. Decisions on the therapeutic modalities are made largely according to the status of tumor growth at the time of diagnosis as well as the expected outcomes of the diseases. Several clinical staging systems have been proposed based on various clinical variables. In general, high-grade and fast growing tumors tend to have a poorer prognosis, while low-grade and slow growing tumors usually associate with a more favorable outcome. For this reason, postoperative recurrence rate and overall survival (OS) in HCC patients are usually used as indicators to evaluate the association between the tissue expression of a molecular factor and the status of HCC cell growth. Such studies could lead to the discovery of clinically applicable prognostic markers, which are pivotal in helping clinicians to improve their decision making in anti-HCC therapies.

MicroRNAs (miRNAs) are non-coding, single stranded RNAs of ∼22 nucleotides, processed from endogenous precursor RNAs with stem-loop structures. They negatively regulate gene expression by targeting mRNAs for cleavage or translational repression [2], and thus play important roles in crucial biological processes, such as organ development, cell differentiation and proliferation, cell apoptosis, and cancer cell invasion [3]. Furthermore, because of their abilities to exert functions similar to tumor suppressors or oncogenic proteins, miRNAs are believed to play an important role in carcinogenesis. To date, distinct expression patterns of miRNA were found to associate with various clinical features as well as patient survivals in many cancers [4]–[8]. Additionally, miRNA expression profiling was found to be a better method to classify poorly differentiated tumors, when compared with mRNA expression profiling [9]. These studies suggested that miRNAs could potentially be developed into a diagnostic or prognostic marker.

Identification of miRNAs differentially expressed between tumors and their non-tumor counterparts is an approach adopted by most researchers. Such experiments have been conducted in HCC by a few groups with inconsistent results. Theoretically, the differentially expressed miRNAs obtained by this strategy should be considered as candidate diagnostic markers, but not prognostic predictors. To identify prognostic predictors, miRNAs differentially expressed in the paraneoplastic liver tissues obtained from HCC patients with known prognosis were searched. To avoid bias resulting from different clinical stages of the study patients, we examined miRNA expression profiles in HCC patients who were all eligible for surgical resection (Barcelona Clinic Liver Cancer [BCLC] stage A). In these patients, all cancerous tissues were surgically removed, resulting in a clinically homogeneous group with no detectable malignant tumors in the liver. Subsequently, the expression levels of miRNAs in the liver tissues were correlated with the time to tumor recurrence. The prognostic predictive value of the candidate miRNA markers were verified in a larger cohort of patients (n = 216). Finally, functional analysis was performed for one of the significant miRNA predictors in two highly tumorigenic hepatoma cell lines to investigate its oncogenic effect.

## Materials and Methods

### Patients and Tissue Specimens

For identifying prognostic miRNA markers, the adjacent noncancerous liver tissues (paraneoplastic tissues) from 228 HCC patients were collected. The noncancerous liver tissues were 1 cm from the tumor site. The clinical records of 228 HCC patients receiving total removal of liver tumors from July 1998 to Aug 2004 in Chang Gung Medical Center, Taiwan were retrieved from Institutional Tissue Bank. Of the 228 patients, 12 with known better and poorer prognosis were subjected for the first-step (pilot) study. Six patients had a recurrence-free survival (RFS) time for more than 5 years (defined as a better prognosis) and six patients had rapid relapse within six-month after operation (defined as a poorer prognosis). It is generally believed that if recurrence or metastasis is not found within five years after the initial treatment of cancer, a complete remission is achieved. Further relapse of HCC rarely occurs (a plateau is reached) if the patients have a recurrence-free survival for more than 5 years. The other 216 HCC patients including 46 females and 170 males were included for subsequent verification analysis. The basic clinical characterization of the 228 patients was listed in Table S1 and S2. The details of sample classification were described in Methods S1. The study protocol was approved by the Medical Ethics and Human Clinical Trial Committee of the Chang Gung Memorial Hospital (IRB No. 100-4016C). Written informed consent was obtained from all patients, and the study was approved by the Ethics Committee of Chang Gung Memorial Hospital.

### Reverse Transcription-quantitative Real-time PCR (RT-qPCR) for miRNA

A stem-loop RT-qPCR method for detection of mature miRNAs was performed according to a previous report [10]. The details of RT-qPCR condition and the quantification software used were described in Methods S1.

### Data Analysis and Statistical Methods

Two normalization methods were used for data analysis in the pilot study. In one method, the raw Ct data were converted to 39-Ct data, which were normalized by the way of global median normalization as previously described [11]. The ΔCt method (the second method) was used to calculated expression level normalized against the miR-30d, which displayed low coefficient of variation (CV = 0.77) and high abundance (mean Ct = 24) in liver tissues in a pilot profiling of the 270 miRNA expression study. The rationale to select these 270 miRNAs from a pool of 472 miRNAs was described in the Methods S1. The miRNA expression level was calculated as POWER(2, ΔCt)×10^6^. For further validation by analyzing a cohort of 216 HCC patients, the ΔCt method was adopted to calculate miRNA expression level. In the pilot study, identification of miRNA markers with significantly differential expression levels between patients with better and poorer prognosis was performed using both Mann-Whitney test and Cox proportional hazard test. Twenty miRNA markers with the lowest *P* values in both tests were included as candidate markers. Associations between miRNA levels and postoperative prognosis were calculated using the Kaplan-Meier method and univariate Cox regression to evaluate the differences of RFS and OS between patients who had high and low expression of candidate miRNAs. The multivariate Cox regression was executed to evaluate the joint effect of confounding factors. The experimental cutoffs were calculated according to the method of minimum *P*-value approach in clinical studies [12]. For each parameter, a series of cutoffs were generated using the following formula: the smallest value + n/10 (the largest value – the smallest value) (n = 1 to 9). The experimental dichotomous groups were thus separated by a cutoff at least 1/10 or at most 9/10 of the factor range. This way of grouping was more readily to be used for making treatment recommendations in the future. The cutoff leading to the smallest *P* value was then selected for subsequent Cox proportional hazard analysis. All statistical analyses were performed with the help of SPSS version 15.0 (Chicago, IL).

### Cell Lines and Lentiviral Transduction

Human HCC cell lines J7 [13] (obtained from Dr. C. S Yang, National Taiwan University, Taiwan) and Mahlavu [14] (obtain from Dr. C. P. Hu, Veterans General Hospital, Taiwan) were maintained in Dulbecco’s modified Eagle’s medium containing 10% fetal calf serum. The inhibited endogenous miR-155 vector (pmiRZip-155 lentivector, SBI System Biosciences, Mountain View, CA) and negative control (pmiRZip lentivector) were transduced into J7 and Mahlavu cell lines using the lentivector expression system. The lentiviral packaging plasmids (pPACKH1 Packaging Plasmid) were purchased from SBI. Packaging of lentiviral expression vector into pseudoviral particles and delivery of packaged lentivector constructs into target cells were performed according to the manufacturer’s procedures. Lentiviral titer was determined by real-time Q-PCR on an ABI 7500 using lentiviral titer kit (MellGen Laboratories Inc., Surrey, BC, Canada). The expression levels of pmiRZip-155 lentivirus expressing anti-miR-155 small RNAs were determined using stem-loop RT-qPCR. The anti-miR-155 primer sequences used were as follows: RT primer, 5′-CTCAACTGGTGTCGTGGAGTCGGCAATTCAGTTGAGTTAATGCT-3′; specific (forward) primer, 5′-CGGCGGGACCCCTATCACGATT-3′; and the universal reverse primer, 5′-CTGGTGTCGTGGAGTCGGCAATTC-3′.

### Assessment of Cell Proliferation and Apoptosis

Cell proliferation was assessed using Thiazolyl Blue Tetrazolium Bromide (MTT, Sigma, St. Louis, MO) assay described previously [15]. Cells apoptosis was evaluated by Terminal Uridine Nick-End Labeling (TUNEL) Assay, where DNA fragmentation was detected using the DeadEndTM Fluorometric TUNEL System (Promega, Madison, WI). The experiments were carried out according to the manufacturer’s procedures.

**Figure 1:**
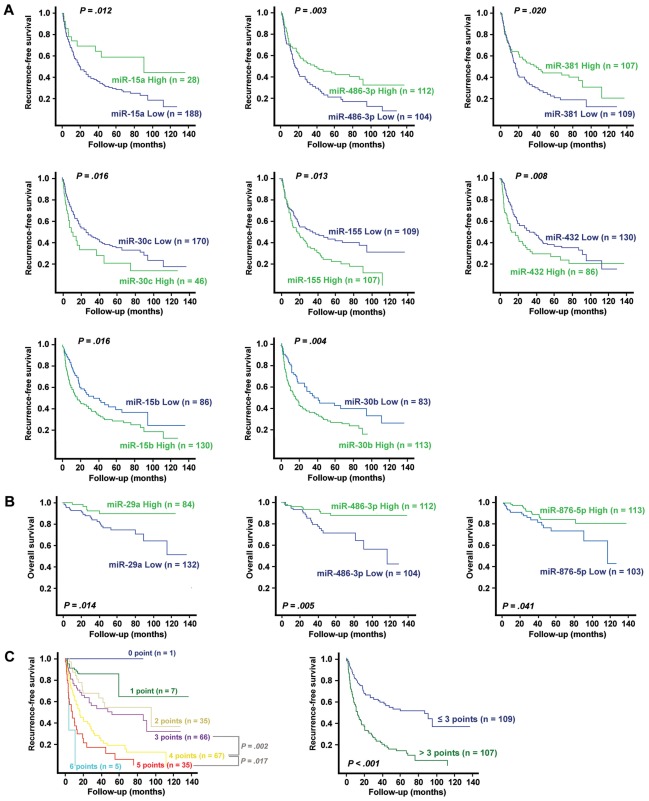
Association between postoperative survivals and expression levels of prognostic miRNAs. (A) Comparison of postoperative RFS between patients with higher and lower expression levels of miR-15a, miR-486-3p, miR-381, miR-30c, miR-155, miR-432, miR-15b and miR-30b, respectively. (B) Comparison of postoperative OS between patients with higher and lower expression levels of miR-29a, miR-486-3p and miR-876-5p. (C) Combination of six miRNA (miR-155, miR-15a, miR-432, miR-486-3p, miR-15b and miR-30b) markers as a prognostic score for RFS in HCC. ‘n’ represents the number of samples in each miRNA marker. The *P* value from the log rank test is shown in each panel.

**Figure 2:**
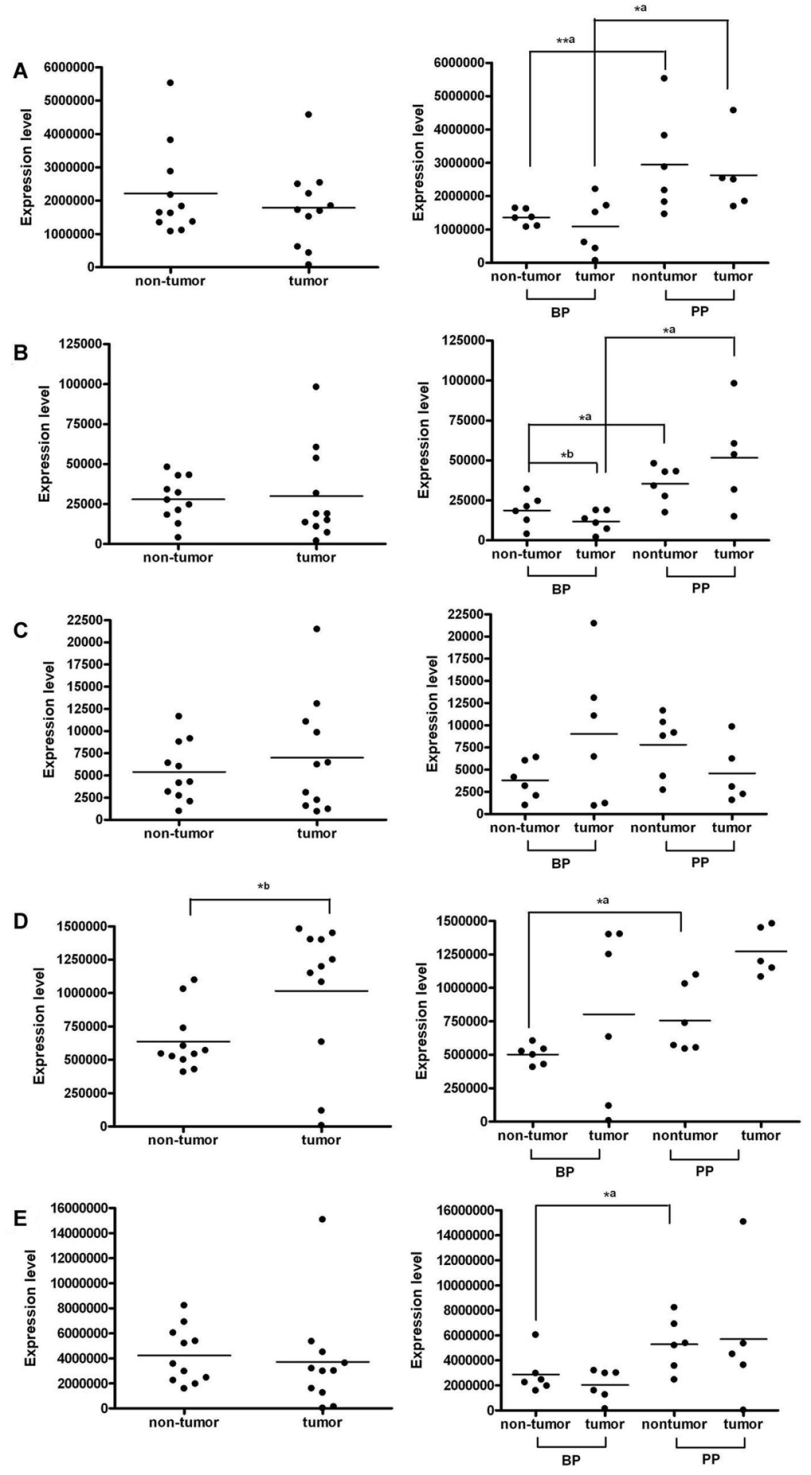
The expression levels of five miRNAs predict a high risk of tumor recurrence in HCC tumor and adjacent non-tumor liver tissues. The expression levels of miR-30c (A), miR-155 (B), miR-432 (C), miR-15b (D), and miR-30b (E) in 11 pairs of HCC tumor and non-tumor liver tissues (to the left). The expression levels of five miRNA in HCC non-tumor liver tissues (BP, n = 6; PP, n = 6) or tumor liver tissue (BP, n = 6; PP, n = 5) with known prognosis (to the right). *, *P*<.05; **, *P*<.01 (*^a^* Wilcoxon Signed Ranks Test; *^b^* Mann-Whitney test).

### Immunoblot Analysis

Proteins were loaded on a 10% SDS-polyacrylamide gel for electrophoresis before being transferred to a PVDF membrane (PerkinElmer, Boston, MA). The membrane was blotted with antibodies to CDK2, cyclin E, CDK4 (Santa Cruz Biotechnology, Santa Cruz, CA), cyclin D1, Bcl-xL, Bcl-2 (Epitomics Inc, Burlingame, CA), or GAPDH (Chemicon, Bedford, MA) for detection. The blots were incubated with horseradish peroxidase conjugated secondary antibody and developed using an ECL detection kit (Millipore Corp., Bedford, MA). The intensities of immunoreactive bands were quantified using Image Gauge software (Fuji Film, Tokyo, Japan) on a densitometer.

### Xenograft Models

Five-week-old, male BALB/cAnN.Cg-*Foxnl^nu^*/CrlNarl nude mice were divided into two groups for subcutaneous injection using J7 (1×10^6^/200 µL PBS) or Mahlavu (1.5×10^6^/200 µL PBS) cells. The cells were infected with either the lentivector expressing antisense miR-155 (n = 6 for J7 xenograft; n = 5 for Mahlavu xenograft) or the control lentivector (n = 6 for J7 xenograft; n = 5 for Mahlavu xenograft). The tumor volume (mm^3^) was measured and calculated using the formula (W^2^×L)/2 (W, the smallest diameter; L, the longest diameter). Mice were sacrificed and the tumors were weighed at day 14 and day 36, respectively, post subcutaneous injection of J7 and Mahlavu cells. All animal experiments were performed in accordance with U.S. National Institutes of Health guidelines, and the Chang Gung Institutional Animal Care and Use Committee Guide for Care and Use of Laboratory Animals. This study was conducted under the approval of Chang Gung Institutionally Animal Care and Use Committee (IACUC Approval No. CGU09-023).

### Immunohistochemistry

Paraffin-fixed xenograft tumor sections (5-µm thick) were prepared for serial xenograft tumor sections and immunohistochemistry was performed to detect Ki-67 as previously described [16].

**Figure 3:**
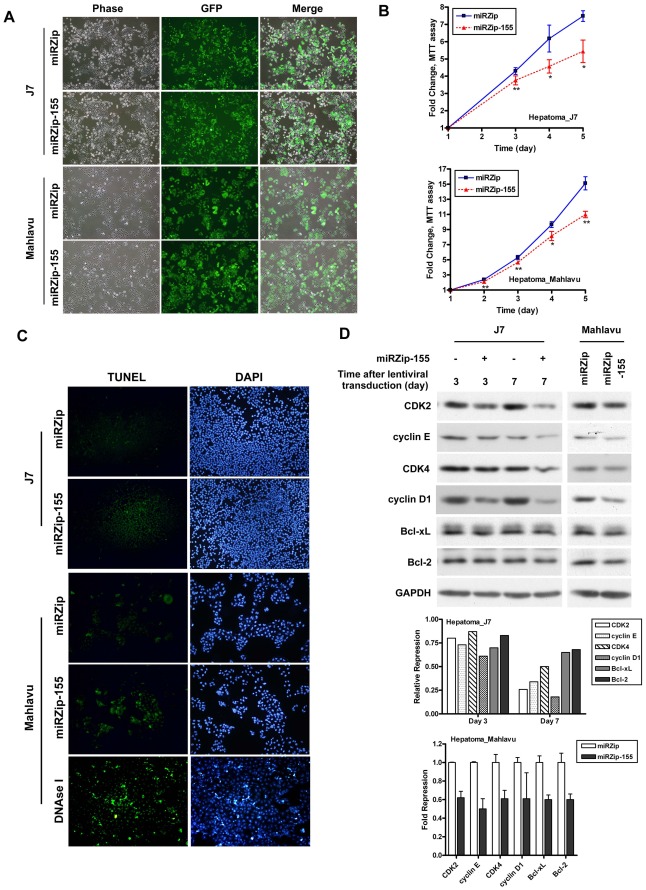
Reduced cell viability by expression knockdown of miR-155 in hepatoma cells. (A) GFP expression in J7 and Mahlavu cells transduced with lentivirus carrying miRZip and miRZip-155 vectors, respectively. GFP, an expression marker encoded by the miRZip vector. (B) Comparisons of the cell proliferation rates assessed by MTT assays between hepatoma cells (J7 [upper panel] and Mahlavu [lower panel]) with (miRZip-155, red triangles) or without (miRZip, blue squares) expression knockdown. *P<.05; **P<.01. (C) Comparisons of cell apoptosis assessed by TUNEL assays between hepatoma cells (J7 [upper panels] and Mahlavu [lower panels]) with (miRZip-155) or without (miRZip) expression knockdown. Cells treated with DNAse I served as a positive control for the TUNEL assay (bottom panel). (D) Immunoblot analysis examined the expression levels of CDK2, cyclin E, CDK4, cyclin D1, Bcl-xL, and Bcl-2 in lentivirus transduced J7 and Mahlavu cells. GAPDH served as a loading control. Quantitative data (by densitometer) were shown in the bottom.

**Figure 4:**
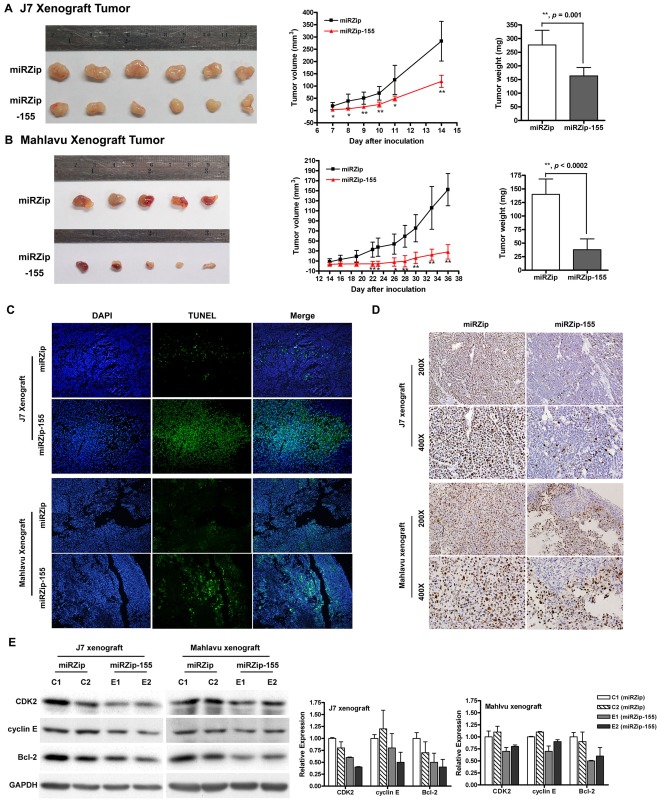
Inhibition of tumor growth by miR-155 expression knockdown in subcutaneous hepatoma xenografts. (A and B) Comparisons of the tumor volumes between hepatoma xenografts with or without miR-155 knockdown. Xenografts were derived from J7 (A) and Mahlavu (B) cells, respectively. Left panels, the final dissected xenograft tumors. Middle panels, comparisons of the growth curves of xenografts. Tumor volumes were measured at days 7 to 14 in J7 xenografts and at days 14 to 36 in Mahlavu xenografts after subcutaneous injection of cells. *P<$>\raster="rg1"<$>.01; **P<$>\raster="rg1"<$>.001; Red triangle, with miR-155 knockdown; Solid squares, without miR-155 knockdown. Right panels, comparisons of the mean tumor weights. Shaded bars, with miR-155 knockdown; Empty bars, without miR-155 knockdown. (C) Comparisons of cell apoptosis assessed by TUNEL assays between xenografts with (miRZip-155) or without (miRZip) miR-155 knockdown. Upper panels, J7 xenografts; Lower panels, Mahlavu xenografts. (D) Comparisons of Ki-67 expression assessed by immunohistochemistry between xenografts with (miRZip-155) or without (miRZip) miR-155 knockdown. Upper panels, J7 xenografts; Lower panels, Mahlavu xenografts. (E) Comparisons of CDK2, cyclin E and Bcl-2 expression levels assessed by immunoblot between xenografts with or without miR-155 knockdown. GAPDH served as a loading control. C1 and C2, xenografts without miR-155 knockdown; E1 and E2, xenografts with miR-155 knockdown. Quantitative data (by densitometer) were shown in the right.

## Results

### Expression Profiles of miRNA in the Paraneoplastic Noncancerous Liver Tissues in Patients with known Better and Poorer Prognosis

As the first step of our study, expression profiles of miRNA in the noncancerous liver tissues from HCC patients with known better (n = 6) and poorer (n = 6) prognosis were analyzed to identify potential prognostic miRNA markers. Twenty miRNA markers with the lowest *P* values in two statistical tests were included as candidate markers. The *P* values ranged from.010 to.055 using Mann-Whitney test and from.004 to.454 using Cox proportional hazard analysis.

### Identification of miRNA Associated with Postoperative Survivals in HCC Patients

In the second step of our study, the adjacent noncancerous liver tissues from 216 HCC patients were used to verify the prognostic predictive value of the 20 miRNAs candidates. Univariate and multivariate analysis was performed to examine the association between the 20 miRNA expression levels and the RFS and OS, respectively (Table S3). Univariate analysis revealed that miR-155, miR-15a, miR-381, miR-432, miR-486-3p, miR-30c, miR-15b and miR-30b were significantly correlated with RFS (*P*≤.021 for all). On the other hand, miR-29a, miR-486-3p and miR-876-5p were associated with OS (*P*≤.046 for all). The effect of these miRNA predictors on HCC RFS was shown using Kaplan-Meier survival analysis (Fig. 1A). The high miR-15a, miR-486-3p, and miR-381 expression significantly predicted a longer RFS (*P*≤.020), while high miR-30c, miR-155, miR-432, miR-15b, and miR-30b expression significantly predicted a shorter RFS (*P*≤.016) (Fig. 1A). In addition, a high expression level of miR-29a, miR-486-3p, and miR-876-5p significantly predicted a longer OS (*P*≤.041) (Fig. 1B). These significant predictors obtained from univariate analysis were included for multivariate Cox proportional analysis. The results showed that miR-155, miR-15a, miR-432, miR-486-3p, miR-15b and miR-30b were the significantly remaining predictors related to RFS (Table S3). When combining these 6 miRNA predictors (miR-155, miR-15a, miR-432, miR-486-3p, miR-15b and miR-30b) to examine the predictive value, it was found that 1 (0.5%), 7 (3.2%), 35 (16.2%), 66 (30.6%), 67 (31.0%), 35 (16.2%), and 5 (2.3%) patients had 0, 1, 2, 3, 4, 5 and 6 unfavorable miRNA factors, respectively. Kaplan-Meier analysis revealed that patients possessing ≤3 and >3 unfavorable miRNA factors constituted two RFS distinguishable subgroups (*P*<.001, Fig. 1C).

### Clinicopathological Factors and miRNA Associated with Postoperative Survivals in HCC

The Cox proportional hazard model was performed to analyze whether the miRNA predictors (miR-155, miR-15a, miR-432, miR-486-3p, miR-15b and miR-30b) were independent factors when clinicopathological variables were included. Univariate analysis revealed that microvascular invasion, tumor number >1, largest tumor diameter >3 cm, ascites, AFP>25 ng/mL, albumin <$>\scale 120%\raster="rg1"<$>4.0 g/dL, prothrombin time >12 seconds, AST >36 U/L, ALT >25 (U/L), higher miR-155 level, lower miR-15a level, higher miR-432 level, lower miR-486-3p level, higher miR-15b level, and higher miR-30b level were significantly associated with a shorter RFS. When these factors were included, multivariate analysis showed that tumor number >1, higher miR-432 level, lower miR-486-3p level, and higher miR-30b level were the significant remaining factors associated with a shorter RFS (Table S4).

### Expression Levels of the Candidate miRNA Predictors between Non-tumor and Tumor Liver Tissues

When these candidate miRNA prognosis predictors were examined, it was found that high expression levels of miR-30c, miR-155, miR-432, miR-15b, and miR-30b were associated with shorter RFS in HCC patients (Fig. 1A), suggesting a possible growth promoting effect in HCCs and thus a candidate anti-HCC target. To further understand the expression levels of these five miRNA predictors in liver tumor tissues of HCC patients with known postoperative prognosis, real-time RT-qPCR was carried out. As shown in figure 2, the expression levels of these markers in the non-cancerous parts were significantly higher in HCC patients with poorer prognosis except for miR-432, in which only borderline significance was observed (*P* = .055) (Fig. 2C, to the right). In particular, the expression levels of miR-30c and miR-155 were also significantly higher in tumor parts in patients with poorer prognosis (PP) (Mann-Whitney test, *P*<.05, Figs. 2A and 2B, to the right). Additionally, the miR-155 expression level was significantly decreased in the tumor parts compared with that in the non-tumor parts of patients with better prognosis (BP) (Wilcoxon rank sum test, *P*<.05). If the prognosis was not taken into consideration, miR-155 expression levels were not significantly different between non-tumor and tumor parts in these HCC patients (Fig. 2B, to the left). Among five miRNAs, only miR-15b (*P*<.05) was found significantly up-regulated in the cancerous tissues (Wilcoxon rank sum test, Fig. 2D, to the left). Therefore, if we only compared the miRNA levels between cancerous and non-cancerous liver tissues (without considering the prognosis), only miR-15b could be identified. These data suggested that comparison of the miRNA expression profiles in the noncancerous liver tissues in HCC patients with known prognosis is a useful strategy to identify miRNA markers which can not be found when only non-tumor and tumor liver tissues were compared.

### Expression Knockdown of miR-155 Reduced Cell Viability in Hepatoma Cells

Of the five miRNAs markers, in which high expression levels were associated with a shorter RFS, only miR-155 displayed significant difference in the tumor parts compared with that in the non-tumor parts of patients with better prognosis, and in both the non-tumor and tumor parts between patients with better and poorer prognosis respectively (Fig. 2B, to the right), thus this marker was selected for functional knockdown study. Two highly tumorigenic hepatoma cell lines, J7 and Mahlavu cells, were transduced with miRZip-155 lentivirus, a lentivirus expressing anti-miR-155 small RNAs (for miR-155 expression knockdown), or empty miRZip (as a mock control). Three days after virus infection, the culture cells expressed high levels of green fluorescent protein (GFP), a marker encoded by the miRZip vector (Fig. 3A). The anti-miR-155 expression levels were measured using anti-miR-155 primers by stem-loop RT-qPCR (Fig. S1). Cell proliferation decreased significantly in miR-155 knockdown J7 and Mahlavu cells, when compared with that in miRZip control cells (Fig. 3B, *P*<.05). The cell cycle related molecules CDK2, CDK4, cyclin E, and cyclin D1 were down-regulated in miR-155 knockdown cells (Fig. 3D). TUNEL assay revealed strong TUNEL labeling in miR-155 knockdown cells. The expression levels of anti-apoptotic related molecules Bcl-xL and Bcl-2 also decreased significantly in miR-155 knockdown cells (Figs. 3C and 3D).

### Expression Knockdown of miR-155 Inhibited Tumor Growth in Hepatoma Xenograft

To understand the effect of miR-155 on tumorigenicity, J7 or Mahlavu cells transduced with miRZip or miRZip-155 lentivirus were injected into nude mice. Palpable tumors were observed in the J7 miRZip group on day 5, while no tumor was found in J7 miRZip-155 group until after day 7. Similarly, palpable tumors were observed in the Mahlavu miRZip group on day 6, while in the Mahlavu miRZip-155 group, tumor was not found until after day 8. All mice of J7 and Mahlavu xenograft models were sacrificed on day 14 and day 36, respectively, to examine the final tumor volume. In addition, the levels of anti-miR-155 expressed in miRZip-155 xenograft tumors were measured using stem-loop RT-qPCR (Fig. S2). The growth rate, and the final tumor volume and weight of the miRZip-155 xenografts were significantly reduced compared with those of the miRZip group in both the J7 and Mahlavu xenograft models (Figs. 4A and 4B). In the miRZip-155 xenograft tumors, more and stronger TUNEL labeling was observed in comparison with that in the miRZip group (Fig. 4C). The expression levels of Ki-67, Bcl-2, CDK2 and cyclin E were significantly decreased (Figs. 4D and 4E). The xenograft experiments clearly indicated that knockdown of endogenous miR-155 suppressed tumor growth.

## Discussion

Comparison of miRNA expression patterns between HCCs and the adjacent noncancerous liver tissues is a generally adopted approach. Previously, expression profiles of miRNA in HCCs have been investigated by several groups. However, the data derived from different studies vary greatly. For example, Murakami et al. analyzed miRNA expression profiles in 24 HCC tissues, 22 adjacent non-tumor tissues, primary cultured hepatocytes and two control cell lines using the microarray methods [17]. Huang et al. using a similar approach to analyze 10 pairs of samples from non-viral hepatitis patients [18]. As a result, completely distinct miRNA expression profiles were reported between the two studies. Only miR-195 was mentioned in both studies, but it was over-expressed in one study and under-expressed in the other. Subsequently, miRNA profiling was conducted by many groups to identify aberrantly expressed miRNAs in HCC, while significant inconsistencies remained [1], [19]–[25]. In a recent study, classification of HCC by miRNA profiling was proposed and miR-517a was identified as an oncogenic miRNA in HCC [26].

Despite the accumulating yet inconsistent data regarding aberrant expression of miRNA in HCC, scanty studies were conducted to illustrate effective prognostic miRNA markers. Some of the aforementioned studies have demonstrated correlation between the aberrantly expressed miRNAs and prognosis [19], [21], [23], [24]. However, the strategy to compare cancerous versus noncancerous tissues was not initially designed as an attempt to identify prognostic markers. Thus, inconsistent results could be expected. In this study, we took a different approach by examining the miRNA expression levels in patients with different postoperative prognosis. Since postoperative recurrent tumors usually arise from the remaining noncancerous liver (after surgical resection), either through de novo oncongenesis or through seeding of micro-clusters of cancer cells, the cellular compositions of the noncancerous liver thus serve as either a source of de novo cancer formation or as a suitable tissue environment for cancer cells seeding and growth. Therefore, it seems reasonable to analyze the molecular compositions of noncancerous tissues for prognostic predictors identification. It could be speculated that aberrant expression of some prognosis related miRNAs in the paraneoplastic tissues were associated with molecular events occurring in early stages of liver cancers. These changes might be masked by other miRNA alterations at certain stages of cancer development and thus could no longer be identified by comparing cancerous and noncancerous tissues thereafter. Actually, in a pilot study, Sato et al. found that the HCC recurrence-related miRNA signature was different in the tumor and non-tumor tissues [24]. Most of the tumor-derived miRNAs were negatively associated with HCC recurrence, while most of the non-tumor-derived miRNAs were positively associated with HCC recurrence. Similarly, in our study, 5 of 8 non-tumor-derived miRNAs (62.5%) were positively associated with recurrence.

After analyzing the expression levels of the 5 miRNAs, in which a higher level was associated with shorter RFS, only miR-15b exhibited significant difference between non-tumor and tumor liver tissues. Actually, in a report by Chung et al. miR-15b was up-regulation in tumor tissue of HCC patients but the up-regulation was associated with a better survival [27]. The reason for this inconsistency was unclear. A possible reason maybe the small number of samples included. In our study, we showed that higher expression of miR-15b predicted a high risk of tumor recurrence through analyzing a larger cohort of HCC patients (n = 217). To clarify the function of miR-15b in HCC, the animal model experiments should be executed in the future. Base on the previous and our present results, the strategy used in this study is capable of identifying new prognostic markers.

During the preparation of this manuscript, aberrant miR-155 expression in HCC was reported in two studies [28], [29]. Han et al. indicated the miR-155 was associated with tumor recurrence and survival in HCC patients following liver transplantation [28]. The result was consistent with our findings in clinicopathologic analysis using non-tumor tissue of HCC patients. In addition, Xie et al. found the ability of miR-155 in enhancement of liver cell tumorigenesis, and suggest miR-155 may be a potential target for HCC therapy [29]. Similarly, in our knockdown miR-155 experiments, the hepatoma cell displayed decrease of cell growth and enhancement of cell death *in vivo* as well as *in vitro*. In fact, expression knockdown of miR-155 was found to increase cell apoptosis in malignant lymphoma in a recent study [30].

Most targeted therapies in anticancer treatments adopt a strategy to utilize an inhibitory molecule to suppress the function of a growth-promoting target. Therefore, we choose a miRNA of which over-expression is associated with shorter survival in this study. Other miRNA predictors which associated with longer RFS might also serve as targets for HCC treatment. For example, researchers found restoration of miR-486 expression resulted in suppression of several pro-oncogenic traits in gastric cancer cell lines, while inhibition of miR-486 expression caused enhanced cell proliferation [31]. A recent study reported loss of chromosome 13q, where miR-15a was located, was associated with shortened cell cycle and increased cell proliferation in dedifferentiated HCC [32]. In addition, the anti-apoptotic protein Bcl-2 is a target of miR-15a in chronic lymphocytic leukemia (CLL), where restoration of miR-15a induces apoptosis in MEG-01 cell line, suggesting a tumor suppressor function of miR-15a [33], [34]. In addition, the OS miRNA predictors (miR-29a, miR-486-3p and miR-876-5p) might also be developed to become the targets for HCC therapy. The miR-29a is believed to be a tumor suppressor in both the lung and pancreatic cancer cell lines and over-expression of miR-29a suppresses invasion and cell proliferation [35]. Another study revealed a growth regulatory effect of miR-29 in human gastric cancer with cell proliferation inhibition and cell cycle arrest [36]. Notably, the identified OS miRNA predictors in this study were all associated with longer OS. Interestingly, in the literature, most OS related miRNA markers such as miR-199b [37], let-7g [38], miR-125b [23], miR-203 [39], miR-199a/b-3p [40], and miR-26 [21] were positively associated with OS in HCC. Only few miRNAs (miR-221 and miR-222) were associated with shorter OS [41], [42].

In conclusion, through analyzing differential expression of miRNAs in HCC patients with different postoperative prognosis, several prognostic miRNA predictors were identified. Among them, miR-155 functioned as an oncomiR in HCC. The present study demonstrated that comparison of differential miRNAs expression in adjacent noncancerous liver tissues is an effective strategy for identifying prognostic markers. These miRNA predictors served as potential targets for anticancer therapy in HCC.

## Supporting Information

Figure S1 The expression levels of anti-miR-155 RNAs in J7 and Mahlavu cells. By use of stem-loop RT-qPCR, the levels of anti-miR-155 RNAs were measured in J7 and Mahlavu cells. The levels of anti-miR-155 RNAs in cells transfected with miRZip (Empty bar) was assigned as 1. The folds of increase of anti-miR-155 in cells transfected with miRZip-155 (Shaded bar) were calculated. Values are mean ± SD. **, *P*<.001.
(EPS)

Figure S2 The expression levels of anti-miR-155 RNAs in J7 (A) and Mahlavu (B) xengraft tumors. By use of stem-loop RT-qPCR, the levels of anti-miR-155 RNAs were measured in J7 and Mahlavu xenografts. The levels of anti-miR-155 RNAs in xenografts transfected with miRZip (Empty bar) was assigned as 1. The folds of increase of anti-miR-155 in xenografts transfected with miRZip-155 (Shaded bar) were calculated. Values were mean ± SD. **, *P*<.001.
(EPS)

Table S1 Basic clinical characterization of 12 patients included for step-1 screening.
(DOC)

Table S2 Basic clinical characterization of 216 patients included for verification test.
(DOC)

Table S3 Univariate and multivariate analysis of the expression levels of 20 candidate miRNA markers for recurrence-free and overall survivals in HCC patients.
(DOC)

Table S4 Univariate and multivariate analysis of clinicopathological and miRNA parameters for recurrence-free survival in HCC patients.
(DOC)

Methods S1 (DOC)
