# Supplementary material for: Identification of Postoperative Prognostic MicroRNA Predictors in Hepatocellular Carcinoma
Source: PLoS One. 2012 May 22;7(5):e37188. doi: 10.1371/journal.pone.0037188 (PMC3358336; doi:10.1371/journal.pone.0037188)
Supplement: Table S1 — Basic clinical characterization of 12 patients included for step-1 screening. (DOC) [file pone.0037188.s003.doc]

**Table S1** Basic clinical characterization of 12 patients included for step-1 screening

| Clinical parameters | Prognosis | |  |
| --- | --- | --- | --- |
|  | Better (n = 6) | Poorer (n = 6) | *P* |
| Age (years) | 49.2 ± 13.7 | 46.5 ± 8.4 | .694 |
| Sex (Male) | 4 | 5 | .999 |
| Cirrhosis | 1 | 5 | .080 |
| HBsAg positive | 5 | 6 | .999 |
| Anti-HCV positive | 1 | 0 | .999 |
| Tumor number |  |  |  |
| 1 | 6 | 3 | .182^a^ |
| 2 | 0 | 0 |  |
| 3 | 0 | 2 |  |
| 4 | 0 | 1 |  |
| Size (Diameter, cm) | 6.6 ± 3.6 | 10.1 ± 5.0 | .199 |
| Microvascular invasion | 0 | 3 | .182 |
| Edmondson’s grading |  |  | .999 |
| 2 | 1 | 0 |  |
| 3 | 4 | 6 |  |
| 4 | 1 | 0 |  |
| Encapsulation | 6 | 5 | .999 |
| Macrovascular invasion | 0 | 0 | .999 |
| Ascites | 0 | 1 | .999 |
| Alpha-fetoprotein (ng/mL) | 850 (3 – 9796)^b^ | 204 (7 – 470) | .462^c^ |
| Albumin (g/dL) | 4.3 ± 0.6 | 3.9 ± 0.6 | .275 |
| Bilirubin (mg/dL) | 4.1 ± 7.1 | 0.8 ± 0.3 | .282 |
| Prothrombin time (sec) | 12.0 ± 0.4 | 13.1 ± 1.0 | .031 |
| Creatinine (mg/dL) | 1.8 ± 1.9 | 0.9 ± 0.2 | .275 |
| AST (U/L) | 57.6 ± 52.1 | 83.2 ± 29.6 | .320 |
| ALT (U/L) | 59.8 ± 49.2 | 53.8 ± 33.8 | .810 |
| Alcoholism | 1 | 1 | .999 |
| Time to recurrence (months) | No recurrence | 2.8 ± 2.2 |  |
| Time to last follow-up (months) | 52.8 ± 5.3 | Not assessed |  |

^a^Comparison between patients with tumor number = 1 and those with tumor number > 1.

^b^Median (range)

^c^Mann-Whitney test
