# Supplementary material for: Identification of Postoperative Prognostic MicroRNA Predictors in Hepatocellular Carcinoma
Source: PLoS One. 2012 May 22;7(5):e37188. doi: 10.1371/journal.pone.0037188 (PMC3358336; doi:10.1371/journal.pone.0037188)
Supplement: Table S2 — Basic clinical characterization of 216 patients included for verification test. (DOC) [file pone.0037188.s004.doc]

**Table S2 Basic clinical characterization of 216 patients included for verification test**

| Clinical parameters | Gender | |  |
| --- | --- | --- | --- |
|  | Female (n = 46) | Male (n = 170) | *P* |
| Age (years) | 55.6 ± 13.4 | 55.0 ± 14.8 | .804 |
| Cirrhosis | 27 (58.7%) | 90 (52.9%) | .597 |
| HBsAg positive | 31 (67.4%) | 133 (78.2%) | .183 |
| Anti-HCV positive | 19 (41.3%) | 36 (21.2%) | .010 |
| Tumor number |  |  | .225^a^ |
| 1 | 28 (60.9%) | 84 (49.4%) |  |
| 2 | 10 (21.7%) | 37 (21.8%) |  |
| 3 | 6 (13.0%) | 35 (20.6%) |  |
| 4 | 2 (4.3%) | 14 (8.3%) |  |
| Size (Diameter, cm) | 6.3 ± 4.9 | 6.8 ± 4.6 | .520 |
| Ascites | 4 (8.7%) | 17 (10.0%) | .791 |
| Alpha-fetoprotein (ng/mL) | 38.5 (3.0 – 327500.0)^b^ | 44.0 (1.5 – 14679.0) | .864^c^ |
| Albumin (g/dL) | 3.8 ± 0.6 | 3.9 ± 0.6 | .317 |
| Bilirubin (mg/dL) | 1.1 ± 1.0 | 1.5 ± 2.1 | .212 |
| Prothrombin time (sec) | 12.0 ± 1.7 | 12.4 ± 1.6 | .139 |
| Creatinine (mg/dL) | 1.2 ± 1.6 | 1.3 ± 1.3 | .661 |
| AST (U/L) | 91.2 ± 104.2 | 86.3 ± 126.9 | .810 |
| ALT (U/L) | 67.4 ± 65.0 | 94.2 ± 156.7 | .259 |
| Alcoholism | 3 (6.5%) | 59 (34.7%) | < .001 |

^a^Comparison between patients with tumor number = 1 and those with tumor number > 1.

^b^Median (range)

^c^Mann-Whitney test
