# Supplementary material for: Identification of Postoperative Prognostic MicroRNA Predictors in Hepatocellular Carcinoma
Source: PLoS One. 2012 May 22;7(5):e37188. doi: 10.1371/journal.pone.0037188 (PMC3358336; doi:10.1371/journal.pone.0037188)
Supplement: Table S3 — Univariate and multivariate analysis of the expression levels of 20 candidate miRNA markers for recurrence-free and overall survivals in HCC patients. (DOC) [file pone.0037188.s005.doc]

**Table S3** Univariate and multivariate analysis of the expression levels of 20 candidate miRNA markers for recurrence-free and overall survivals in HCC patients

| miRNA level^a^ | No. of patients | Recurrence-free survival | |  | Overall survival | |
| --- | --- | --- | --- | --- | --- | --- |
|  |  | Hazard Ratio (95% CI) | Adjusted Hazard Ratio (95% CI) |  | Hazard Ratio (95% CI) | Adjusted Hazard Ratio (95% CI) |
| miR-151 |  |  |  |  |  |  |
| Low | 121 |  |  |  |  |  |
| High | 95 | 1.004 (0.697 – 1.446) |  |  | 0.741 (0.346 – 1.589) |  |
| miR-345 |  |  |  |  |  |  |
| Low | 159 |  |  |  |  |  |
| High | 57 | 0.806 (0.525 – 1.239) |  |  | 0.313 (0.095 – 1.033) |  |
| miR-374 |  |  |  |  |  |  |
| Low | 40 |  |  |  |  |  |
| High | 176 | 1.302 (0.817 – 2.007) |  |  | 0.734 (0.323 – 1.669) |  |
| miR-197 |  |  |  |  |  |  |
| Low | 122 |  |  |  |  |  |
| High | 94 | 0.977 (0.678 – 1.407) |  |  | 0.694 (0.323 – 1.490) |  |
| miR-29a |  |  |  |  |  |  |
| Low | 132 |  |  |  |  |  |
| High | 84 | 0.839 (0.576 – 1.221) |  |  | 0.319 (0.122 – 0.833)^p^ | 0.402 (0.146 – 1.105) |
| miR-155 |  |  |  |  |  |  |
| Low | 109 |  |  |  |  |  |
| High | 107 | 1.577 (1.097 – 2.266)^b^ | 2.002 (1.324 – 3.027)^j^ |  | 0.823 (0.394 – 1.719) |  |
| miR-15a |  |  |  |  |  |  |
| Low | 188 |  |  |  |  |  |
| High | 28 | 0.459 (0.246 – 0.856)^c^ | 0.478 (0.248 – 0.920)^k^ |  | 0.387 (0.092 – 1.630) |  |
| miR-381 |  |  |  |  |  |  |
| Low | 109 |  |  |  |  |  |
| High | 107 | 0.653 (0.455 – 0.938)^d^ | 0.701 (0.436 – 1.129) |  | 0.543 (0.258 – 1.143) |  |
| miR-432 |  |  |  |  |  |  |
| Low | 130 |  |  |  |  |  |
| High | 86 | 1.625 (1.130 – 2.337)^e^ | 1.816 (1.203 – 2.740)^l^ |  | 1.279 (0.614 – 2.661) |  |
| miR-486-3p |  |  |  |  |  |  |
| Low | 104 |  |  |  |  |  |
| High | 112 | 0.585 (0.407 – 0.841)^f^ | 0.543 (0.330 – 0.893)^m^ |  | 0.343 (0.157 – 0.752)^q^ | 0.502 (0.218 – 1.154) |
| miR-30c |  |  |  |  |  |  |
| Low | 170 |  |  |  |  |  |
| High | 46 | 1.713 (1.098 – 2.673)^g^ | 1.115 (0.705 – 1.763) |  | 1.258 (0.514 – 3.084) |  |

**Table S3** Continued

| miRNA level^a^ | No. of patients | Recurrence-free survival | |  | Overall survival | |
| --- | --- | --- | --- | --- | --- | --- |
|  |  | Hazard Ratio (95% CI) | Adjusted Hazard Ratio (95% CI) |  | Hazard Ratio (95% CI) | Adjusted Hazard Ratio (95% CI) |
| miR-101 |  |  |  |  |  |  |
| Low | 99 |  |  |  |  |  |
| High | 117 | 1.223 (0.852 – 1.755) |  |  | 1.039 (0.507 – 2.130) |  |
| miR-15b |  |  |  |  |  |  |
| Low | 86 |  |  |  |  |  |
| High | 130 | 1.080 (1.014 – 1.151)^h^ | 1.074 (1.002 – 1.152)^n^ |  | 1.034 (0.913 – 1.170) |  |
| miR-22 |  |  |  |  |  |  |
| Low | 108 |  |  |  |  |  |
| High | 108 | 0.939 (0.655 – 1.344) |  |  | 0.938 (0.458 – 1.925) |  |
| miR-30b |  |  |  |  |  |  |
| Low | 83 |  |  |  |  |  |
| High | 133 | 1.101 (1.031 – 1.175)^i^ | 1.102 (1.025 – 1.185)^o^ |  | 1.009 (0.890 – 1.143) |  |
| miR-34c-3p |  |  |  |  |  |  |
| Low | 103 |  |  |  |  |  |
| High | 113 | 1.049 (0.732 – 1.505) |  |  | 1,300 (0.624 – 2.709) |  |
| miR-129-5p |  |  |  |  |  |  |
| Low | 91 |  |  |  |  |  |
| High | 125 | 0.813 (0.565 – 1.169) |  |  | 1.110 (0.527 – 2.338) |  |
| miR-186 |  |  |  |  |  |  |
| Low | 113 |  |  |  |  |  |
| High | 103 | 1.065 (0.744 – 1.524) |  |  | 0.610 (0.290 – 1.285) |  |
| miR-196b |  |  |  |  |  |  |
| Low | 84 |  |  |  |  |  |
| High | 132 | 0.829 (0.573 – 1.198) |  |  | 0.535 (0.260 – 1.100) |  |
| miR-876-5p |  |  |  |  |  |  |
| Low | 103 |  |  |  |  |  |
| High | 113 | 0.773 (0.539 – 1.107) |  |  | 0.474 (0.228 – 0.986)^r^ | 0.510 (0.243 – 1.073) |

^a^The cutoffs of high and low expression levels were determined according to the method describe in Materials and methods.

^b^*P* = .014; ^c^*P* = .014; ^d^*P* = .021; ^e^*P* = .010; ^f^*P* = .004; ^g^*P* = .018; ^h^*P* = .017; ^i^*P* = .004;

^j^*P* = .001; ^k^*P* = .027; ^l^*P* = .015; ^m^*P* = .016; ^n^*P* = .043; ^o^*P* = .009;

^p^*P* = .020; ^q^*P* = .008; ^r^*P* = .046.

For other comparisons, *P* > .05.
