# Supplementary material for: Identification of Postoperative Prognostic MicroRNA Predictors in Hepatocellular Carcinoma
Source: PLoS One. 2012 May 22;7(5):e37188. doi: 10.1371/journal.pone.0037188 (PMC3358336; doi:10.1371/journal.pone.0037188)
Supplement: Table S4 — Univariate and multivariate analysis of clinicopathological and miRNA parameters for recurrence-free survival in HCC patients. (DOC) [file pone.0037188.s006.doc]

**Table S4** Univariate and Multivariate Analysis of Clinicopathological and miRNA Parameters for Recurrence-Free Survival in HCC Patients

| Parameter |  | No. of patients | Mean RFS, months (95% CI) | HR (95% CI) | Adjusted HR (95% CI) |
| --- | --- | --- | --- | --- | --- |
| Age (years) | ≦ 60 | 125 | 52.9 (39.4 – 61.1) |  |  |
|  | ＞ 60 | 91 | 44.8 (34.9 – 54.6) | 0.925 (0.638 – 1.341) |  |
| Gender | Female | 46 | 59.3 (41.4 – 77.3) |  |  |
|  | Male | 170 | 48.9 (37.1 – 56.2) | 1.377 (0.873 – 2.171) |  |
| Cirrhosis | No | 99 | 56.7 (43.1 – 70.3) |  |  |
|  | Yes | 117 | 41.4 (33.5 – 51.8) | 1.241 (0.862 – 1.786) |  |
| Alcoholism | No | 154 | 50.9 (40.8 – 61.0) |  |  |
|  | Yes | 62 | 46.9 (33.0 – 60.8) | 1.115 (0.758 – 1.640) |  |
| Tumor characteristics | |  |  |  |  |
| Microvascular invasion | No | 135 | 57.3 (46.2 – 68.4) |  |  |
|  | Yes | 81 | 32.5 (23.3 – 41.6) | 1.765 (1.223 – 2.544)* | 1.178 (0.787 – 1.765) |
| Edmondson’s grading | I-II | 69 | 50.9 (35.6 – 66.2) |  |  |
|  | III-IV | 147 | 48.8 (38.8 – 58.8) | 1.116 (0.750 – 1.660) |  |
| Encapsulation | No | 58 | 36.4 (25.3 – 47.6) |  |  |
|  | Yes | 158 | 54.0 (43.7 – 64.3) | 0.789 (0.533 – 1.168) |  |
| Tumor number | 1 | 112 | 58.7 (46.6 – 70.7) |  |  |
|  | ＞ 1 | 104 | 34.2 (26.3 – 42.3) | 1.556 (1.082 – 2.238)* | 1.596 (1.070 – 2.381)* |
| Largest tumor size (diameter, cm) | ≦ 3 | 60 | 62.8 (48.3 – 77.2) |  |  |
|  | ＞ 3 | 156 | 43.1 (33.5 – 52.7) | 1.809 (1.181 – 2.771)* | 1.431 (0.912 – 2.246) |
| Macrovascular invasion | No | 175 | 51.1 (41.6 – 60.6) |  |  |
|  | Yes | 41 | 45.9 (30.9 – 60.9) | 1.023 (0.638 – 1.639) |  |
| Ascites | No | 195 | 52.4 (43.2 – 61.5) |  |  |
|  | Yes | 21 | 25.7 (9.2 – 42.3) | 1.996 (1.158 – 3.441)* | 1.491 (0.812 – 2.740) |
| Serology |  |  |  |  |  |
| AFP (ng/mL) | ≦ 25 | 96 | 57.5 (44.4 – 70.7) |  |  |
|  | ＞ 25 | 120 | 43.2 (32.7 – 53.7) | 1.622 (1.114 – 2.361)* | 1.567 (1.036 – 2.368) |
| Albumin (g/dL) | ≦ 4.0 | 129 | 42.0 (31.4 – 52.7) |  |  |
|  | ＞ 4.0 | 87 | 59.7 (47.0 – 72.4) | 0.598 (0.411 – 0.868)* | 0.723 (0.477 – 1.094) |

**Table S4** Continued

| Parameter |  | No. of patients | Mean RFS, months (95% CI) | HR (95% CI) | Adjusted HR (95% CI) |
| --- | --- | --- | --- | --- | --- |
| Bilirubin (mg/dL) | ≦ 1.2 | 152 | 54.3 (44.0 – 64.6) |  |  |
|  | ＞ 1.2 | 64 | 37.0 (25.6 – 48.3) | 1.366 (0.922 – 2.023) |  |
| Prothrombin time (sec) | ≦ 12 | 101 | 60.0 (46.6 – 73.3) |  |  |
|  | ＞ 12 | 115 | 41.3 (31.2 – 51.4) | 1.493 (1.038 – 2.148)* | 1.436 (0.959 – 2.151) |
| Creatinine (mg/dL) | ≦ 1.0 | 117 | 47.0 (37.0 – 57.0) |  |  |
|  | ＞ 1.0 | 99 | 54.7 (40.9 – 68.4) | 0.917 (0.637 – 1.319) |  |
| AST (U/L) | ≦ 36 | 86 | 66.1 (52.4 – 79.8) |  |  |
|  | ＞ 36 | 130 | 39.8 (30.3 – 49.3) | 1.896 (1.285 – 2.796)* | 1.325 (0.837 – 2.095) |
| ALT (U/L) | ≦ 25 | 37 | 72.6 (52.0 – 93.3) |  |  |
|  | ＞ 25 | 179 | 45.3 (36.4 – 54.1) | 2.193 (1.207 – 3.982)* | 1.287 (0.658 – 2.518) |
| Anti-HCV | Negative | 161 | 50.5 (40.4 – 60.6) |  |  |
|  | Positive | 55 | 44.1 (32.6 – 55.6) | 0.901 (0.592 – 1.371) |  |
| HBsAg | Negative | 52 | 47.8 (30.5 – 65.0) |  |  |
|  | Positive | 164 | 50.6 (40.8 – 60.4 ) | 0.957 (0.622 – 1.471) |  |
| miRNA variables |  |  |  |  |  |
| miR-155 | Low | 109 | 62.3 (49.3 – 75.2) |  |  |
|  | High | 107 | 35.8 (26.9 – 44.8) | 1.577 (1.097 – 2.266)* | 1.497 (0.976 – 2.296) |
| miR-15a | Low | 188 | 43.7 (35.5 – 51.9) |  |  |
|  | High | 28 | 81.5 (56.4 – 106.6) | 0.459 (0.246 – 0.856)* | 0.511 (0.259 – 1.008) |
| miR-432 | Low | 130 | 53.8 (43.7 – 63.9) |  |  |
|  | High | 86 | 42.1 (28.4 – 55.8) | 1.625 (1.130 – 2.337)* | 1.580 (1.005 – 2.484)* |
| miR-486-3p | Low | 104 | 35.9 (26.2 – 45.6) |  |  |
|  | High | 112 | 63.9 (51.0 – 76.9) | 0.585 (0.407 – 0.841)* | 0.447 (0.290 – 0.688)* |
| miR-15b | Low | 86 | 60.3 (45.0 – 75.7) |  |  |
|  | High | 130 | 42.3 (32.6 – 51.9) | 1.080 (1.014 – 1.151)* | 1.073 (0.998 – 1.154) |
| miR-30b | Low | 83 | 64.7 (49.8 – 79.7) |  |  |
|  | High | 133 | 36.0 (28.8 – 43.1) | 1.101 (1.031 – 1.175)* | 1.091 (1.011 – 1.177)* |

**P* < .05

HR, hazard ratio; CI, confidence interval
