# Supplementary material for: Identification of Postoperative Prognostic MicroRNA Predictors in Hepatocellular Carcinoma
Source: PLoS One. 2012 May 22;7(5):e37188. doi: 10.1371/journal.pone.0037188 (PMC3358336; doi:10.1371/journal.pone.0037188)
Supplement: Methods S1 — (DOC) [file pone.0037188.s007.doc]

**Supplementary Methods**

**Patients and tissue specimens**

All samples were frozen to –70^o^C, immediately after surgical resection. The following clinicopathological data were retrospectively reviewed: age, gender, presence of liver cirrhosis, HBsAg positive, anti-HCV positive, number of tumors, largest tumor size, microvascular invasion, Edmondson’s histologic grade, presence of tumor capsule, macrovascular invasion, presence of ascites upon surgery, alpha-fetoprotein (AFP), albumin, bilirubin, prothrombin time, creatinine, aspartate aminotransferase (AST), alanine aminotransferase (ALT), alcohol usage, date of tumor recurrence, and date of last follow-up or HCC related death. In our medical center, patients with main portal vein thrombosis were excluded from surgical management. Minor portal vein invasion (secondary branch) discovered during or after surgery was categorized as macrovascular invasion.

**Reverse transcription-quantitative real-time PCR (RT-qPCR) for miRNA**

Briefly, 10 μl RT reaction mixture containing miRNA-specific stem-loop RT primers (final concentration, 2 nM each), 500 μM dNTP, 0.5 μl Superscript III (Invitrogen, Carlsbad, CA), 0.5 μl RNaseOut (Invitrogen), and 1 μg total RNA was used for RT reaction performed at 16°C for 30 min, followed by 50 cycles of reaction at 20°C for 30 s, 42°C for 30 s, and 50°C for 1 s. The RT products were diluted 20-fold before submitted for qPCR. The details of qPCR were described previously . Briefly, 0.5 μl of diluted RT product was used as template in a 6 μl PCR reaction mixture, which contained 1× SYBR Master Mix (Applied Biosystem, Foster City, CA), 200 nM miRNA-specific forward primer, and 200 nM universal reverse primers. The condition for qPCR was 95°C for 10 min, followed by 40 cycles of reaction at 95°C for 15 s and 63°C for 32 s. All qPCR reactions were performed on the ABI 7900HT Fast Real-Time PCR system (Foster City, CA). The ABI 7900HT SDS 2.3 software was used to calculate the threshold cycle (Ct) and relative quantification. The Ct was defined as the cycle number at which fluorescence was determined to be statistically significant above background. The sensitivity and dynamic range of the stem-loop RT-qPCR method were assessed using standard SYBR green assay for the 270 miRNA markers used in this study . Twenty-five miRNAs were excluded for further study because of low abundance (Ct > 35) in the 12-sample analysis. MiR-30d was used for tissue RNA normalization.

**Selection of the 270 miRNA markers from a pool of 472 miRNAs**

To perform an effective survey for clinically applicable prognostic miRNA marker, a total of 472 miRNAs were subjected for preliminary screening to eliminate those with extremely low levels in normal tissues. Such miRNAs with low abundance could cause difficulty in the quantitative assessment. A mixture of total RNA derived from 20 types of normal tissues was generated. To identify miRNAs which were detectable in normal tissues, the mixture of total RNAs was divided into two aliquots. In one experiment, reverse transcriptase (RTase) was removed from the reverse transcription reaction for qPCR, of which the Ct value was defined as the background (B). In the other experiment, reverse transcription was performed with RTase for qPCR, in which the Ct value was defined as a signal (S). Of the 472 miRNAs, those with △Ct value < 5 from Ct_S_ − Ct_B_, and those with Ct value < 33 were eliminated. Finally, 270 miRNAs were selected for further focused screening.

**Reference**

1. Chen HC, Chen GH, Chen YH, Liao WL, Liu CY, et al. (2009) MicroRNA deregulation and pathway alterations in nasopharyngeal carcinoma. Br J Cancer 100: 1002-1011.
